# Patellar instability MRI measurements are associated with knee joint degeneration after reconstruction of the medial patellofemoral ligament

**DOI:** 10.1007/s00256-021-03832-6

**Published:** 2021-07-04

**Authors:** Paula Giesler, Frederic A. Baumann, Dominik Weidlich, Dimitrios C. Karampinos, Matthias Jung, Christian Holwein, Julia Schneider, Alexandra S. Gersing, Andreas B. Imhoff, Fabian Bamberg, Pia M. Jungmann

**Affiliations:** 1 Department of Diagnostic and Interventional Radiology, Medical Center – University of Freiburg, Faculty of Medicine, University of Freiburg, Hugstetter Strasse 55, 79106 Freiburg, Germany; 2 Clinical and Interventional Angiology, University Hospital of Zurich, University of Zurich, Raemistrasse 100, 8091 Zurich, Switzerland; 3 Department of Diagnostic and Interventional Radiology, Technical University of Munich, Ismaninger Strasse 22, 81675 Munich, Germany; 4 Department of Orthopaedic Sports Medicine, Technical University of Munich, Ismaninger Strasse 22, 81675 Munich, Germany; 5 Department of Neuroradiology, University Hospital, LMU Munich, Marchioninistraße 15, 81377 Munich, Germany

**Keywords:** Magnetic resonance imaging, Ligaments, Knee, Cartilage, Risk factors, Patellofemoral joint, Joint instability, Patellar dislocation

## Abstract

**Objective:**

To qualitatively and quantitatively evaluate the 2-year magnetic resonance imaging (MRI) outcome after MPFL reconstruction at the knee and to assess MRI-based risk factors that predispose for inferior clinical and imaging outcomes.

**Materials and methods:**

A total of 31 patients with MPFL reconstruction were included (22 ± 6 years, 10 female). MRI was performed preoperatively in 21/31 patients. Two-year follow-up MRI included quantitative cartilage T2 and T1rho relaxation time measurements at the ipsilateral and contralateral knee. T2_relative_ was calculated as T2_patellofemoral_/T2_femorotibial_. Morphological evaluation was conducted via WORMS scores. Patellar instability parameters and clinical scores were obtained. Statistical analyses included descriptive statistics, t-tests, multivariate regression models, and correlation analyses.

**Results:**

Two years after MPFL reconstruction, all patellae were clinically stable. Mean total WORMS scores improved significantly from baseline to follow-up (mean difference ± SEM, − 4.0 ± 1.3; P = 0.005). As compared to patients with no worsening of WORMS subscores over time (n = 5), patients with worsening of any WORMS subscore (n = 16) had lower trochlear depth, lower facetal ratio, higher tibial-tuberosity to trochlear groove (TTTG) distance, and higher postoperative lateral patellar tilt (P < 0.05). T2_relative_ was higher at the ipsilateral knee (P = 0.010). T2_relative_ was associated with preoperatively higher patellar tilt (P = 0.021) and higher TTTG distance (P = 0.034). TTTG distance, global T2 values, and WORMS progression correlated with clinical outcomes (P < 0.05).

**Conclusion:**

MPFL reconstruction is an optimal treatment strategy to restore patellar stability. Still, progressive knee joint degeneration and patellofemoral cartilage matrix degeneration may be observed, with patellar instability MRI parameters representing particular risk factors.

**Supplementary Information:**

The online version contains supplementary material available at 10.1007/s00256-021-03832-6.

## Introduction

Single and recurrent lateral patella dislocation is associated with gradual cartilage deterioration on MR imaging and increases the risk for knee joint osteoarthritis [1–3]. Reconstruction of the medial patellofemoral ligament (MPFL) is a successful treatment for patellar instability with restoration of the deficient primary medial patellar soft tissue restraint and good functional outcomes [4–6]. Although patella stabilization is reported to result in lower redislocation rates than conservative treatment [5–9], a higher rate of patellofemoral osteoarthritis was reported after surgical treatment [10]. However, the authors state that these findings should be interpreted with great caution due to publication bias, methodological quality of the evidence base, and variety surgical interventions [10].

MR imaging-based trochlear dysplasia parameters are associated with early knee joint osteoarthritis in patients without MPFL reconstruction [11, 12]. It remains unclear whether MPFL reconstruction contributes to the development of osteoarthritis. In a retrospective study, morphological changes on postoperative MR imaging after MPFL reconstruction were described [13]. There is no MR imaging study that assesses the qualitative or the quantitative knee joint degeneration after MPFL reconstruction.

Quantitative T2 and T1rho relaxation time measurements are able to non-invasively assess the biochemical composition of cartilage, correlate with cartilage matrix degeneration, and predict morphological cartilage loss and osteoarthritis [14–17]. Increasing cartilage T2 relaxation times primarily correlate with decreasing collagen contents, collagen disruption, and increasing water contents while increasing T1rho relaxation times correlate with decreasing proteoglycan contents and increasing water contents [18]. Early cartilage matrix degeneration may be depicted in young individuals without morphological cartilage defects who are at risk for early osteoarthritis [11, 14, 15].

The purposes of this study were to qualitatively and quantitatively evaluate the 2-year MRI outcome after MPFL reconstruction at the knee and to assess MRI-based risk factors that predispose for inferior clinical and imaging outcomes.

## Subjects and methods

### Subjects

The study was approved by the local Institutional Review Board (Ethikkommission Technical University of Munich, Germany). All patients gave written informed consent prior to participation in the study. Patients were recruited between 11/2015 and 06/2017. Inclusion criteria were MPFL reconstruction at the knee 2 years prior of inclusion to this study and age ≥ 18 years at the follow-up time-point. Exclusion criteria were previous surgery, concomitant surgery, or follow-up surgery, as well as MRI contraindications such as claustrophobia, MRI-incompatible implants (e.g., pacemaker), or pregnancy. Further exclusion criteria were insufficient MR image quality or insufficient or incomplete MRI data. A detailed flowchart illustrating the patient selection is provided in the supplemental material S1.

### Surgery

Isolated MPFL reconstruction was performed by specialized orthopedic sports surgeons as previously described [19–21]. In brief, an anatomical double-bundle technique with an autologous gracilis tendon was applied. At the patellar insertion, two bioresorbable anchors were inserted. At the femoral insertion, one bioresorbable interference screw was inserted under fluoroscopic guidance [22]. The graft was finally fixed at 30° of flexion to ensure an optimal contact pressure. Postoperatively, knee flexion was limited to 90°. Passive motion and partial weight-bearing with 20 kg were required for the initial 2 weeks after surgery. For the duration of the hospital stay, continuous passive movement was applied for daily knee flexion exercises. After week 2, weight-bearing loads were continuously increased until full weight-bearing was achieved 6 weeks postoperatively. After week 6, gradual increases of the range of motion were encouraged until full range of motion was aimed.

### Clinical outcome

Two years after MPFL reconstruction, all knee joints were examined regarding patellofemoral stability. The Kujala anterior knee pain scoring system [23] and the Knee Injury and Osteoarthritis Outcome Score (KOOS) were used to semi-quantitatively assess clinical symptoms and function [24, 25]. The Kujala score contains a 13-item questionnaire for the patient-reported assessment of anterior knee pain. It assesses the ability to perform several activities and the presence of symptoms/disabilities. The range of the Kujala score is 0 to 100 with 100 representing an optimal score (no knee problems) and 0 representing the worst score [25]. The KOOS score assesses pain, symptoms, activities of daily living, sports and recreation function, and knee-related quality of life. The KOOS score is provided as a percentage value with 100% representing an optimal score (no knee problems) and 0% representing the worst score [24].

### Magnetic resonance imaging

MR imaging of the knee joint was performed at a 3-T MR scanner (Ingenia, Philips Healthcare, Best, the Netherlands) using a dedicated transmit-receive 16-channel knee coil (Medical Advances, Milwaukee, WI, USA). Preoperatively, morphological MR imaging was performed at the ipsilateral knee. Two years after MPFL reconstruction, morphological and quantitative MR imaging was performed at the ipsilateral knee and at the contralateral knee for intrapersonal comparisons of quantitative relaxation time measurements of the articular cartilage. At the ipsilateral knee, morphological pulse sequences included two-dimensional (2D) intermediate-weighted (IM-w) turbo spin echo (TSE) sequences in three planes and sagittal T1-w TSE sequences [26]. At the contralateral knee, for morphological evaluation, only sagittal IM-w TSE sequences were acquired due to scan-time restrictions. At both knees, sagittal 2D T2 multi-slice multi-echo (MSME) SE sequences included six echoes at echo times (TE) = 10, 20, 30, 40, 50, and 60 ms [26]. For sagittal three-dimensional (3D) T1rho relaxation time measurements, a rotary echo spin-lock pulse was implemented as a preparation module in a three-dimensional spoiler gradient echo sequence for acquisition of T1rho-weighted data with a spin-lock frequency of 500 Hz at spin-lock times (SLT) of 10, 20, 40, 60, and 80 ms [27]. Detailed MR imaging parameters are provided in Table 1.

**Table 1:** MR imaging pulse sequence parameters

Sequence	T1-w TSE	IM-w TSE	IM-w TSE	IM-w TSE	MESE T2	SPGR T1rho
Additional features	2D	2D, blade, FS	2D, blade, FS	2D, blade, FS	2D	3D, FS
Plane	Sagittal	Sagittal	Coronal	Transverse	Sagittal	Sagittal
Echo time (TE; ms)	13	44	44	40	10, 20, 30, 40, 50, 60	-
Spin lock duration (TSL; ms)	-	-	-	-	-	10, 20, 40, 60, 80
Repetition time (TR; ms)	785	4202	3363	5456	2200	9.6
Field of view (FOV; mm)	140	140	140	150	140	140
In-plane resolution (mm^2^)	0.4 × 0.4	0.4 × 0.4	0.4 × 0.4	0.4 × 0.4	0.4 × 0.4	0.5 × 0.5
Slice thickness (mm)	3	3	3	3	2.5	2.0
Number of slices	28	30	24	36	30*	45*
Slice distance (mm)	3.6	3.6	3.6	3.3	2.5	2
Flip angle (°)	90	90	90	90	90	10
Bandwidth per pixel (Hz)	143	187	187	201	251	217
Phase encoding direction	Column	Column	Column	Row	Column	Row
Number of averages	1	2	2	2	1	1
Acquisition time (min)	3:06	4:50	4:50	5:42	5:33	11:00

MR, magnetic resonance; FOV, field of view; w, weighted; IM, intermediate; FS, fat-saturated; MESE, multi-slice multi-echo spin-echo; SPGR, spoiled gradient recalled. *Per echo.

### Semi-quantitative MR assessment

MR images were reviewed by Picture Archiving Communication System (PACS) workstations (Easy Vision, Philips, Best, Netherlands). Morphological assessment of the clinical MR images regarding early osteoarthritic changes was conducted via the Whole-Organ Magnetic Resonance Imaging Score of the knee (WORMS) pre- and postoperatively by two readers in consensus (P.M.J. and A.S.G., 12 and 8 years of experience in musculoskeletal imaging) [28]. For each subregion, the structures were assessed as the following (supplemental material S2, adjusted from [26]): (i) meniscus (score 0–4 in 6 regions), (ii) ligaments (score 0–4 in 6 locations), (iii) cartilage (score 0–6 in 6 regions), (iv) bone marrow (score 0–3 in 6 regions), (v) flattening or depression of articular surfaces (score 0–3 in 6 regions), (vi) subarticular cysts (score 0–3 in 6 regions), (vii) osteophytes (score 0–3 in 6 regions), and (v) other abnormalities (effusion (score 0–3), intraarticular body (score 0–2), baker cyst (score 0–3)). The total WORMS score was calculated as a sum score of all subscores similar to previous publications [26, 29], resulting in a range of 0 to 164. The higher the score, the more pathological changes were present [28].

### Patellar instability measurements

Trochlear dysplasia and patellar alignment measurements were performed by one investigator (F.A.B.), supervised by one specialized musculoskeletal radiologist (P.M.J., 12 years of experience in musculoskeletal imaging). Trochlear dysplasia parameters including the medial-to-lateral trochlear facetal ratio, the trochlear depth, and the sulcus angle were obtained at the axial slice 30 mm proximal to the knee joint line as previously described [11, 30, 31]. Patellar alignment parameters included measurements for patellar height, patellar tilt, and patellar maltracking. Patella height was determined via the Caton-Dechamps index [31, 32]. The Caton-Dechamps index was calculated by dividing the distance of the lowest point of the patellar articular surface and the anterior point of the lateral tibial plateau by the patellar articular length as measured on sagittal MR images [33, 34]. Lateral patellar tilt was determined by calculating the angle between a reference line through the patella and a line tangential along the femoral condyles posteriorly as measured on transverse MR images [31]. Patellar maltracking was determined by measuring the tibial-tuberosity to trochlear groove (TTTG) distance between the center of the tibial tuberosity and the deepest point of the trochlear groove on a line parallel to the line tangential along the femoral condyles posteriorly [35–37]. Presence of a localized, nipple-like anterior trochlear prominence at the most anterior and proximal part of the femoral trochlea on midsagittal MR images ≥ 7 mm was noted [30]. Measurements on MR images are illustrated in supplemental material S3.

### Quantitative MR image assessment

For the postprocessing of T1rho images, an in-house developed algorithm programmed in Matlab (Mathworks, Natick, MA, USA) by one MR physicist specialized in musculoskeletal imaging (D.W., 3 years of experience), supervised by a senior MR physicist specialized in musculoskeletal imaging (D.C.K., 12 years of experience), was used. T2 and T1rho maps were reconstructed by fitting the images pixel by pixel using a Levenberg–Marquardt mono-exponential non-negative least squares fit algorithm [38]. The first echo (TE = 10 ms) was excluded from the fitting algorithm to minimize the effects from stimulated echo signals on the calculated relaxation times. One investigator (J.S.) performed manual segmentation of cartilage in 6 different compartments (patella, trochlea, medial femoral condyle (MFC), lateral femoral condyle (LFC), medial tibia plateau (MT), lateral tibia plateau (LT)) on all knee MR images as described previously [39] and was supervised by one experienced musculoskeletal radiologist (P.M.J., 12 years of experience). Relative values were calculated as T2_relative_ = [(T2_patella_ + T2_trochlea_)/2] / [(T2_MFC_ + T2_LFC_ + T2_MT_ + T2_LT_)/4] and T1rho_relative_ = [(T1rho_patella_ + T1rho_trochlea_)/2] / [(T1rho_MFC_ + T1rho_LFC_ + T1rho_MT_ + T1rho_LT_)/4], respectively.

### Reproducibility

Good intra- and interreader reproducibility was shown previously in our research group [26]. Intra-class correlation coefficients (ICCs) for WORMS subscores ranged between 0.81 and 0.88. Intra- and interreader agreement for cartilage relaxation time measurements was high. The root mean square error coefficient of variation for T2 was between 0.98 and 1.72% for intrareader agreement and between 1.12 and 2.51% for interreader agreement.

### Statistical analysis

Statistical analysis was performed using SPSS v26 (IBM, Armonk, NY, USA; P.G. and P.M.J.). As level of significance 0.05 was assumed for all tests. Using Kolmogorov–Smirnov’s tests, normal distribution was confirmed for the main outcome parameters. For paired analyses within the same subjects, paired t-tests were applied. For comparisons of cases with worsening of any WORMS subscore during follow-up versus cases with no worsening of any WORMS subscore during follow-up, unpaired t-tests were applied. Multivariate linear regression models adjusting for the main risk factors for osteoarthritis, including age, gender, and body mass index (BMI), were additionally used to confirm the independence of the results from these risk factors. Means ± standard deviation (SD), mean differences between groups ± standard error of the mean (SEM), and lower and upper 95% confidence intervals (95% CI) were calculated. Unilateral Spearman’s rank correlations adjusting for age, gender, and BMI were obtained for correlation analyses. Finally, multivariate linear regression models with forward progression were applied for identification of the most relevant patellar instability parameters.

## Results

### Subjects

Finally, 31 patients with isolated unilateral MPFL reconstruction were included in this study. In the analyzed cohort (14 male, 17 female), the mean ± SD age at surgery was 22 ± 6 years (range 14–37 years). The mean follow-up time was 27 ± 5 months (2.3 ± 0.4 years). There were 17 right knees and 14 left knees treated with MPFL reconstruction. The mean BMI was 23.5 ± 4.1 kg/m^2^. In 22 patients, chronic patellar instability was diagnosed. Eight patients had traumatic patellar dislocations.

### Morphological MR imaging

At follow-up, the mean ipsilateral total WORMS score was 6.2 ± 6.2 (n = 31) and the mean contralateral total WORMS score was 1.7 ± 0.5 (P < 0.001). Mean cartilage sum scores, patellar cartilage subscores, BME sum scores, patellar BME subscores, and baker cyst scores at ipsilateral knees were significantly higher than those at contralateral knees (P < 0.05). All other subscores were not significantly different.

### Change of morphological scores

In n = 21 cases, preoperative MR images were available that were performed at a maximum of 6 weeks before surgery. Mean total WORMS scores improved significantly from baseline to follow-up (total WORMS at baseline, 10.3 ± 8.2; mean difference ± SEM, − 4.0 ± 1.3, P = 0.005; n = 21). In 5/21 cases, no worsening was observed for any subscore. Worsening of any WORMS subscore was observed in 16/21 patients; some of these patients showed improvements in other scores, finally resulting in no increase of total WORMS scores. Worsening of WORMS scores was mainly due to worsening of patellar cartilage subscores (7/21; P = 0.766) and patellar osteophyte subscores (6/21; P = 0.010). Significant improvements were observed for BME subscores: All BME at the lateral femoral condyle improved from baseline to follow-up (n = 12 cases, P < 0.001); BME was reduced, but still present at follow-up in 2/12 of these cases. BME at the patella newly occurred in n = 3 cases, increased in 1/10 cases with BME at baseline while decreasing in 9/10 cases (P = 0.010). BME at the trochlea was reduced or disappeared in all 3/21 cases with BME at the trochlea at baseline (P = 0.110). Significant improvement was also found for joint effusion (P = 0.004). For the other subscores, the differences were not significant (P > 0.05).

### Quantitative MR imaging

Two years after MPFL surgery, absolute cartilage T2 and T1rho values at the global knee were similar at the ipsilateral knee and at the contralateral knee (global T2, mean ± SEM: 31.1 ± 0.6 ms versus 31.7 ± 0.6 ms, P = 0.179; global T1rho: 39.5 ± 0.4 ms versus 39.5 ± 0.5 ms, P = 0.886, Table 2). Also at the patella, absolute T2 and T1rho values showed no significant difference between the ipsilateral knee and the contralateral knee (P > 0.05). T2_relative_ at the ipsilateral knee was significantly higher than T2_relative_ at the contralateral knee (1.10 ± 0.12 versus 1.05 ± 0.12; P = 0.010). For T1rho_relative_, the difference was not significant (1.30 ± 0.02 versus 1.26 ± 0.02, P = 0.075). In Spearman’s rank correlations, higher postoperative patellar cartilage scores correlated significantly with higher T2_relative_ (R = 0.416, P = 0.028) but not with T1rho_relative_ (R = 0.183, P = 0.353).

**Table 2:** Means ± SEM for the ipsilateral and contralateral cartilage T2 and T1rho relaxation times of the global knee and of the different knee compartments. For the differences between ipsilateral and contralateral values, confidence intervals (lower 95% confidence interval (CI), upper 95% CI) and P-values are provided. Relative values were calculated as patellofemoral mean value divided by femorotibial mean value. T2_relative_ was significantly higher at the ipsilateral knee

Compartment	T2 (ms) ipsilateral	T2 (ms) contralateral	95% CI	P	T1rho (ms) ipsilateral	T1rho (ms) contralateral	95% CI	P
Global knee	31.1 ± 0.6	31.7 ± 0.6	(− 1.4, 0.3)	0.179	39.5 ± 0.4	39.5 ± 0.5	(− 0.7, 0.8)	0.886
Relative value	1.10 ± 0.16	1.05 ± 0.12	(0.01, 0.09)	0.010*	1.30 ± 0.02	1.26 ± 0.02	(− 0.00, 1.85)	0.075
Patella	33.5 ± 0.6	33.5 ± 0.7	(− 1.3, 1.4)	0.939	46.6 ± 0.8	45.8 ± 0.9	(− 0.8, 2.3)	0.316
Trochlea	32.5 ± 0.7	31.9 ± 0.7	(− 0.8, 2.1)	0.363	46.7 ± 1.2	45.6 ± 1.0	(− 1.2, 3.3)	0.348
Medial femoral condyle	34.1 ± 0.7	34.0 ± 0.8	(− 2.4, 0.5)	0.205	41.8 ± 0.8	40.8 ± 0.9	(− 0.7, 2.5)	0.238
Lateral femoral condyle	34.5 ± 0.8	35.6 ± 0.8	(− 2.4, 0.2)	0.089	42.1 ± 0.6	41.5 ± 0.9	(− 1.2, 2.4)	0.503
Medial tibia plateau	26.5 ± 0.6	27.7 ± 0.8	(− 2.2, − 0.1)	0.030	30.3 ± 1.0	32.0 ± 0.8	(− 4.1, 0.7)	0.156
Lateral tibia plateau	25.6 ± 0.6	26.6 ± 0.7	(− 1.7, − 0.2)	0.020	29.7 ± 0.9	31.1 ± 0.7	(− 3.3, 0.6)	0.172

*P < 0.05.

### Clinical outcome

**Fig. 1:**
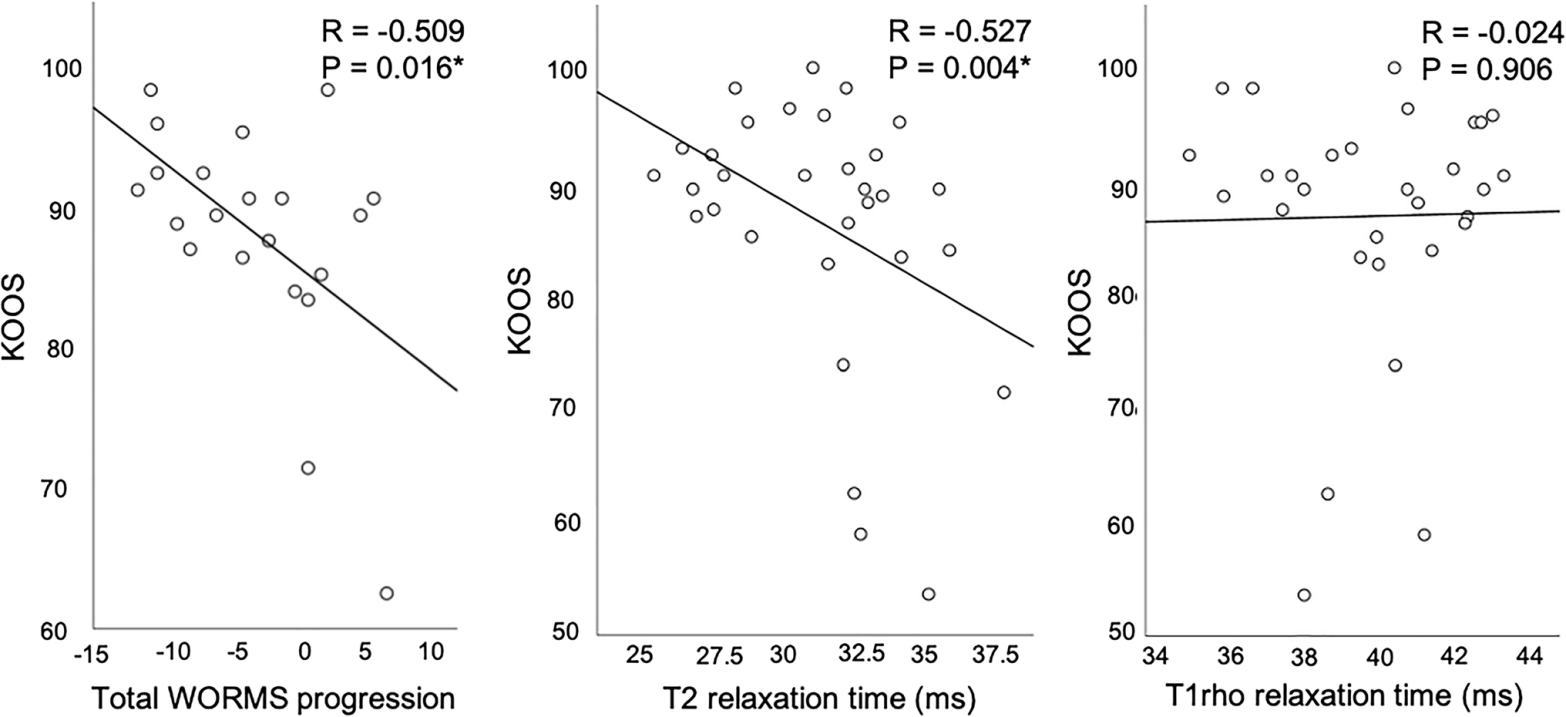
Bivariate linear fit of KOOS scores by total WORMS progression between baseline and follow-up, T2 relaxation times, and T1rho relaxation times in patients with MPFL reconstruction. *P < 0.05

Two years after MPFL reconstruction, patellar stability was restored in all individuals. No patient suffered from redislocation during the observation time. The mean ± SD postoperative KOOS score was 86.4 ± 11.3 (range 53.6 to 100.0). The mean ± SD Kujala score was 82.8 ± 13.3 (range 56.0 to 100.0). In Spearman’s rank correlations, more severe total WORMS progression over time correlated significantly with worse clinical outcomes (n = 21; KOOS: R =  − 0.509, P = 0.016; Kujala: R =  − 0.335, P = 0.087; Fig. 1). Higher global cartilage T2 values correlated significantly with worse clinical outcomes (lower KOOS score: R =  − 0.527, P = 0.004; Kujala: R =  − 0.626, P < 0.001). Clinical scores did not correlate significantly with T1rho values (P > 0.05). When implementing total WORMS at follow-up, total WORMS progression, global T2 values, and global T1rho values in a multivariate linear regression model, the outcome parameters total WORMS progression (B =  − 0.51, P = 0.014) and global T2 values (B =  − 0.39, P = 0.049) were most predictive for clinical outcomes (KOOS).

### Patellar instability parameters

Means ± SD for patellar instability measurements are provided in Table 3. Comparisons between ipsilateral and contralateral patellar instability measurements are provided in the supplemental material S4. The contralateral knee showed significantly deeper trochleae and lower TTTG distances (P < 0.05). The other parameters were not significantly different. None of the patients had a nipple-like prominence on knee MRI.

**Table 3:** Spearman correlations of different patellar instability MR imaging measurements and cartilage T2 and T1rho relaxation time measurements. Covariates were age, gender, and body mass index

Parameter	Mean ± SD	Correlation with global T2	Correlation with global T1rho	Correlation with T2_relative_	Correlation with T1rho_relative_
Facetal ratio	0.49 ± 0.12	R = 0.306; P = 0.113	R = 0.096; P = 0.634	R = − 0.062; P = 0.752	R = 0.041; P = 0.835
Trochlear depth (mm)	4.7 ± 1.4	R = 0.233; P = 0.233	R = − 0.068; P = 0.738	R = 0.019; P = 0.923	R = 0.083; P = 0.676
Sulcus angle (°)	149 ± 11°	R = − 0.088; P = 0.657	R = 0.022; P = 0.914	R = 0.003; P = 0.990	R = − 0.003; P = 0.987
TTTG (mm)	13.3 ± 4.0	R = − 0.105; P = 0.596	R = 0.312; P = 0.113	R = 0.403; P = 0.034*	R = 0.097; P = 0.622
Patellar tilt preOP (°)	16.9 ± 9.6	R = − 0.076; P = 0.765	R = 0.267; P = 0.300	R = 0.538; P = 0.021*	R = 0.145; P = 0.567
Patellar tilt postOP (°)	13.16.2	R = − 0.254; P = 0.191	R = 0.204; P = 0.308	R = 0.137; P = 0.488	R = 0.117; P = 0.554
Caton-Dechamps preOP	1.15 ± 0.04	R = 0.062; P = 0.807	R = − 0.473; P = 0.055	R = − 0.102; P = 0.688	R = − 0.138; P = 0.584
Caton-Dechamps postOP	1.13 ± 0.02	R = − 0.142; P = 0.471	R = − 0.111; P = 0.582	R = − 0.189; P = 0.335	R = − 0.279; P = 0.150

TTTG, tibial-tuberosity to trochlear groove distance; MR, magnetic resonance; preOP, preoperatively; postOP, postoperatively; SD, standard deviation

*P < 0.05

**Fig. 2:**
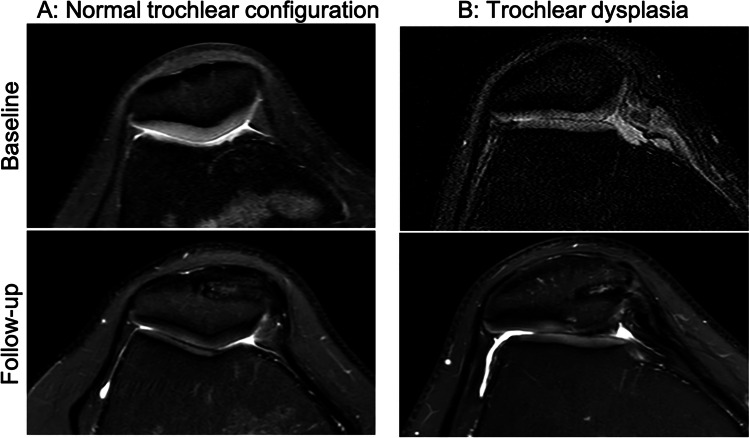
Trochlear dysplasia as a risk factor for progressive knee joint degeneration. Column **A**: Patient with normal trochlear configuration and without morphological cartilage defects at baseline and at follow-up after MPFL reconstruction. Column **B**: Patient with trochlear dysplasia and with no patellar cartilage defect at baseline but with new patellar morphological cartilage loss at follow-up after MPFL reconstruction. All images are transverse intermediate weighted turbo spin echo sequences with fat saturation

**Fig. 3:**
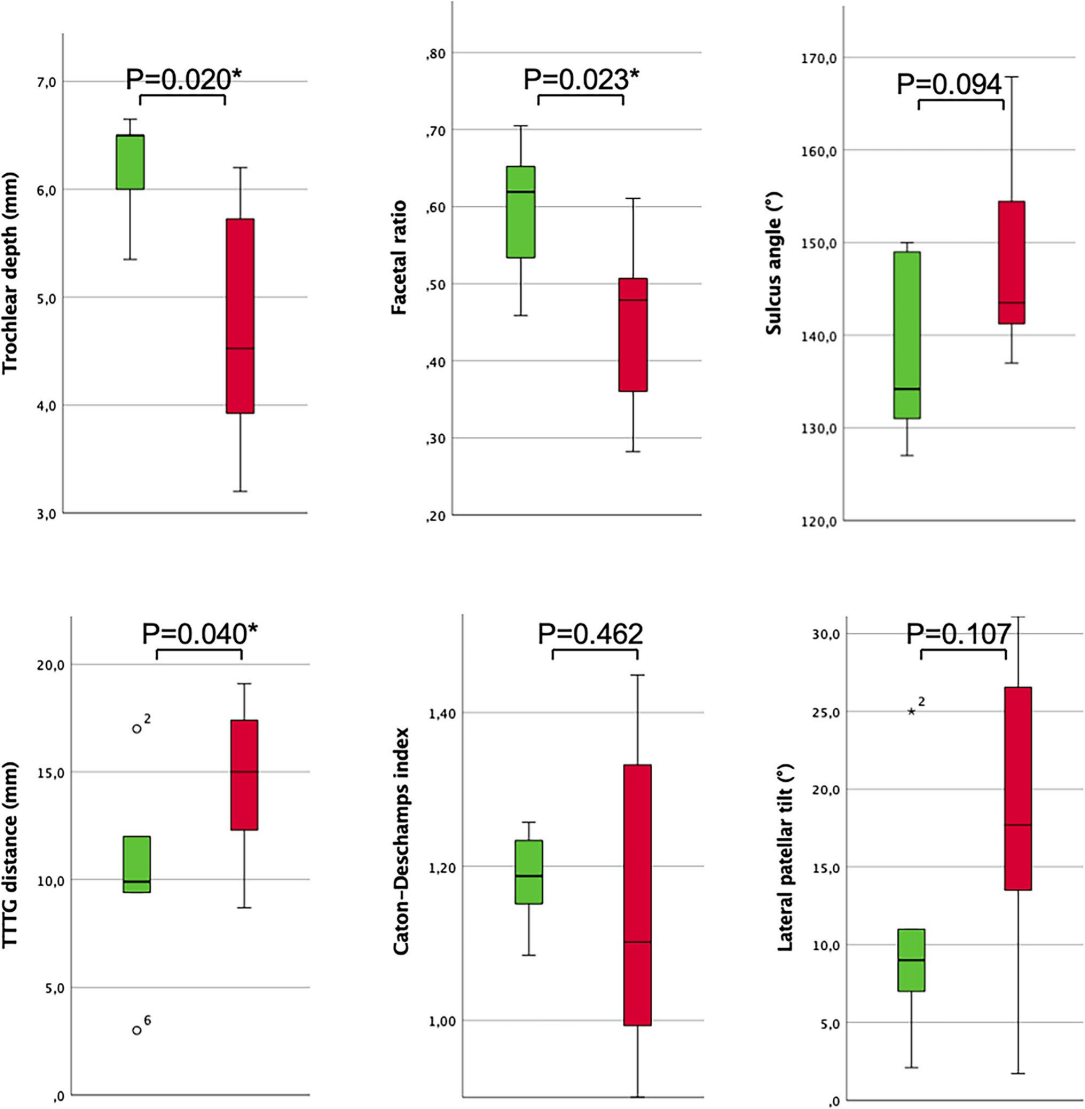
Comparison of the mean preoperative MR imaging-based patellar instability and trochlear dysplasia values between patients without progression of knee joint degeneration from baseline to follow-up (n = 5, depicted in green) and patients with progression of any WORMS subscore, indicating progression of knee joint degeneration from baseline to follow-up 2.3 years after MPFL reconstruction (n = 16, depicted in red). Average values, upper and lower boxes indicating the 2 and 3 quartile and upper and lower whisker indicating the range of the parameters. *P < 0.05

As compared to patients with no worsening of WORMS subscores over time (n = 5), patients with worsening of any WORMS subscore (n = 16) had more pathological patellar instability values (Figs. 2 and 3): Patients with worsening of WORMS subscores showed a lower trochlear depth (mean ± SEM; 4.4 ± 0.4 mm versus 6.2 ± 0.2 mm, P = 0.020), a lower medial-to-lateral trochlear facetal ratio (0.44 ± 0.13 versus 0.59 ± 0.10, P = 0.023), a higher TTTG distance (14.5 ± 0.8 mm versus 10.3 ± 2.3 mm, P = 0.040), and a higher postoperative lateral patellar tilt (13.9 ± 1.1° versus 5.7 ± 2.7°; P = 0.003). The difference for sulcus angle showed a statistical trend (147 ± 2° versus 138 ± 5°, P = 0.094). Significances persisted after adjustment for covariates. Patients with worsening of patellar cartilage lesions during follow-up presented a significantly lower trochlear depth (3.9 ± 0.8 mm versus 5.3 ± 0.3 mm, P = 0.046) and a larger sulcus angle (151 ± 5° versus 141 ± 2°, P = 0.039).

**Fig. 4:**
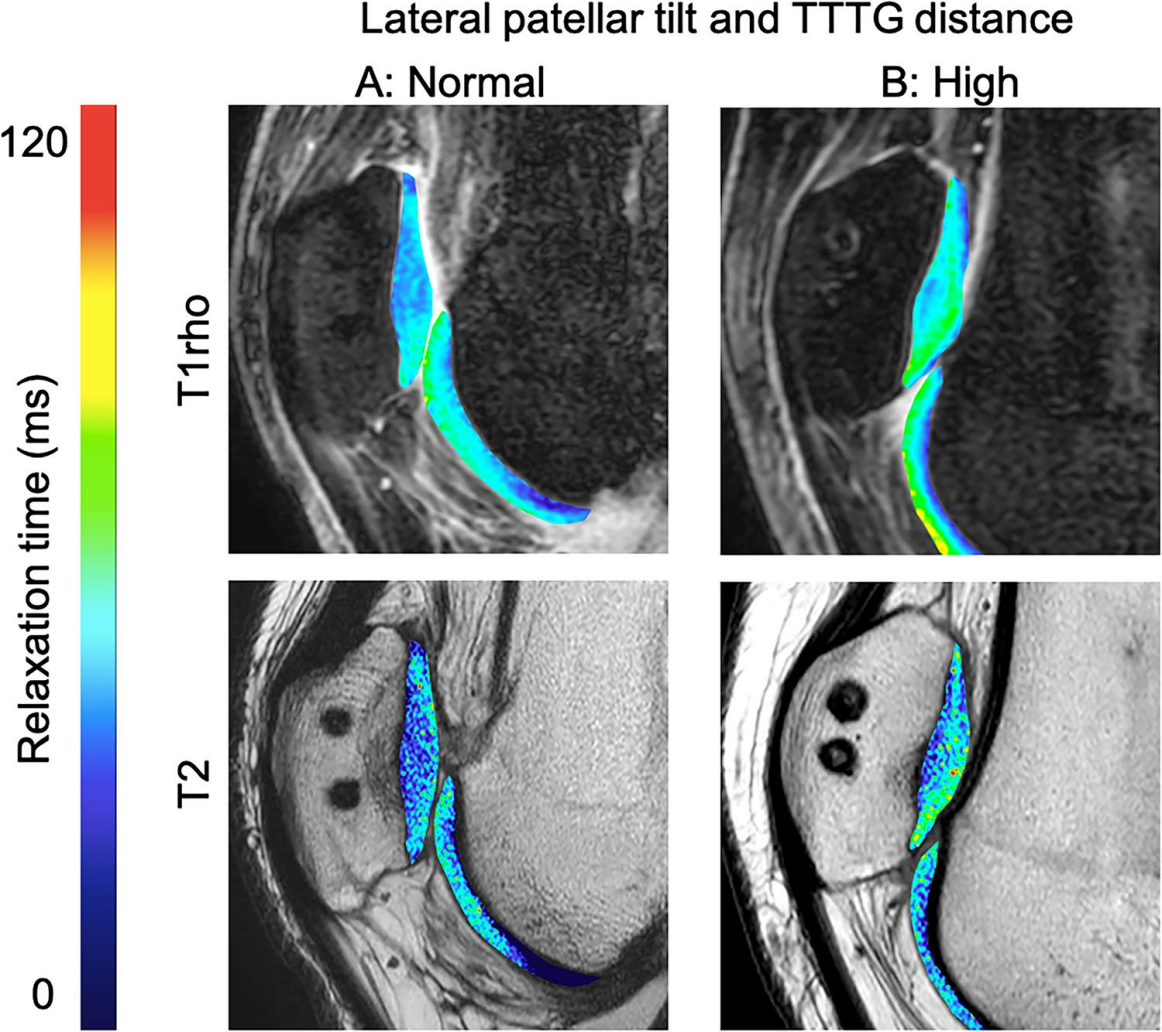
T1rho (upper row) and T2 (lower row) color maps of the patellar cartilage overlaid on the first-echo images. Blue color indicates low and red color high cartilage relaxation times. Subjects with normal preoperative patellar tilt and normal TTTG distance (column **A**) showed lower cartilage relaxation times than subjects with high preoperative patellar tilt and high TTTG distance (column **B**)

Higher postoperative T2_relative_ correlated significantly with preoperatively increased lateral patellar tilt (R = 0.538, P = 0.021; Table 2) and with a higher TTTG distance (R = 0.403; P = 0.034; Fig. 4). For the other patellar instability measurements, there was no significant correlation with quantitative relaxation times (P > 0.05). Lateral patellar tilt was normalized after MPFL surgery (16.9 ± 2.1° versus 11.9 ± 1.3, P = 0.018, n = 21).

### Multivariate regression

When implementing MR imaging-based patellar instability measurements in multivariate linear regression models, the parameters postoperative lateral patellar tilt (B = 0.55, P = 0.003) and trochlear depth (B =  − 0.41, P = 0.022) showed the most relevant influence on any WORMS progression. Preoperatively increased lateral patellar tilt had the most relevant influence on T2_relative_ (B = 0.53, P = 0.013). The TTTG distance (B = 2.15, P = 0.041) was most predictive for clinical outcomes (KOOS).

## Discussion

The present study describes the quantitative and the longitudinal semi-quantitative MR imaging outcome in patients 2 years after isolated MPFL reconstruction at the knee. An overall favorable outcome after MPFL reconstruction was achieved. Global quantitative T1rho and T2 relaxation times of articular cartilage were similar between the ipsilateral operated joint and the contralateral non-operated joint. However, T2_relative_ was increased at the ipsilateral knee, indicating early cartilage matrix degeneration at the patellofemoral joint. Preoperative patellar tilt and maltracking (high TTTG distance) were identified as risk factors for high T2_relative_. Despite MPFL reconstruction, the majority of patients showed minor progressive morphological degenerative changes at the knee joint during longitudinal follow-up. These patients showed MR imaging parameters indicating trochlear dysplasia: (i) a more shallow trochlea, (ii) a higher trochlear facetal asymmetry, (iii) by trend a larger sulcus angle as well as (iv) maltracking and (v) postoperatively higher lateral patellar tilt. A shallow trochlea, increased lateral patellar tilt and maltracking were identified as the most relevant parameters. Following, MPFL reconstruction seems to be an optimal treatment strategy to restore clinical patellar stability. Still, progressive knee joint degeneration and increased patellofemoral cartilage matrix degeneration may be observed. Additional treatment of trochlear dysplasia and tibial tubercle transfer in the case of borderline anatomic shapes may need to be discussed.

The overall good outcome after MPFL reconstruction with restored function and improving WORMS scores over time is in line with previous studies that assessed clinical outcomes after isolated MPFL reconstruction [40–42]. Clinical KOOS and Kujala scores are similar to previously published outcomes [43–45]. In the present study, worse clinical scores were significantly associated with progressive knee joint degeneration and higher global T2 values. Earlier studies also reported that cartilage relaxation times at the knee joint correlated with clinical scores [46–48]. Still, patellofemoral T2 was increased at the ipsilateral knee as compared to the contralateral knee, indicating early cartilage matrix degeneration.

In the literature, several MR imaging-based measurements have been described to be associated with patellar instability and trochlear dysplasia [31]. Increased prevalent and progressive patellofemoral degeneration was reported for patients with pathological patellar alignment, patellar tilt, and trochlear dysplasia measurements [11, 37, 49–53]. It was reported that by normalizing patellar tracking, patellofemoral contact pressure and joint stress may be decreased [54–59]. Potentially, the stress reduction is inferior in those patients with persistent joint abnormalities. It was previously reported that trochlear dysplasia is the most common anatomic risk factor for patellar redislocation after primary isolated MPFL reconstruction followed by increased maltracking, while tunnel positioning had no significant influence [20, 40]. In the present study, besides trochlear dysplasia parameters and TTTG distance, postoperatively persistent high lateral patellar tilt was identifyed as a risk factor for progressive knee joint degeneration after surgery. In contrast, preoperative lateral tilt and TTTG distance were identified as risk factors for early patellofemoral cartilage matrix degeneration after MPFL reconstruction. Therefore, it may be suspected that the matrix degeneration observed in our study was at least partially preexistent before surgery. Longitudinal MR imaging assessments have previously suggested that altered patellar alignment is associated with prevalence and progression of degenerative changes in asymptomatic subjects, in patients with patellofemoral pain, and in patients with anterior cruciate ligament (ACL) reconstruction [36, 60–62]. In earlier quantitative MR imaging studies, patellar malposition was associated with increased patella T1rho values [63, 64]. In normal controls and patients with patellofemoral instability, Kim et al. found a correlation between TTTG distance and patella T2 values [65]. In contrast, no significant correlation between TTTG distance and patella T2* relaxation times was found in soccer players [66]. T1rho and T2 values may be more sensitive to detect early cartilage matrix changes than T2* values [67, 68]. Based on these studies, although isolated MPFL reconstruction may be appropriate in most patients, the indication for additional trochlear surgery such as deepening trochleoplasty and tibial tubercle transfer in borderline cases may need to be considered [21, 40, 69].

There are only few studies assessing patients after MPFL reconstruction via MR imaging postoperatively [13, 69]. One study that used pre- and postoperative morphological MR imaging assessed 20 patients with MPFL reconstruction and deepening trochleoplasty in a combined surgery. They mainly describe the normalization of patellar alignment including patellar tilt, as also shown in our study [69]. They do not assess degenerative changes [69]. In a retrospective study, Wong et al. concluded that the appearance of the MPFL graft itself is not an influencing factor for progression of patellofemoral osteoarthritis [13]. The high rate of progressive morphological cartilage defects (48%) and the lack of the influence of patellar instability measurements in that study may be due to the inhomogeneity of the cohort, the retrospective selection bias, and the non-longitudinal outcome parameters assessed [13]. The present study is the first to systematically assess quantitative cartilage matrix degeneration after MPFL reconstruction and the first to assess longitudinal progression of degenerative changes by concentrating on a specific postoperative follow-up time-point.

Correlations of cartilage T1rho and T2 values with knee joint degeneration and with cartilage degeneration were described earlier [61, 70]. However, it is important to underline the need for multimodal MR imaging in a complementary approach as done in this study [71–73], since relaxation time measurements may be limited once advanced cartilage defects occur [15]. In our study, correlation of quantitative cartilage relaxation times with clinical and morphological scores and measurements was only found for T2 values, indicating that these may be more reliable than T1rho values.

There are several limitations of this study. First, the sample size was rather small and there was no control group. Results need to be confirmed in larger cohort studies with long-term follow-ups. Second, only patients aged > 18 years at follow-up were included in this study. Since patellofemoral dislocations are common in teenagers, this may represent a bias. Still, 10 patients were < 18 years at the time of surgery. Third, quantitative T2 and T1rho relaxation times were only acquired at the postoperative time-point and it remains unclear whether the cartilage matrix changes were preexisting. Still, assessment of longitudinal changes was conducted via semi-quantitative WORMS scores. Fourth, only patients with isolated MPFL reconstruction were included. Fifth, since patellar instability and trochlear dysplasia are often found bilateral, side-to-side comparisons are of questionable value. However, trochlear depth and TTTG distance were significantly worse at the ipsilateral knee in our cohort. Last, due to scan-time restrictions, contralateral morphological images were only acquired in the sagittal plane and were reformatted for further analyses. This may have reduced the accuracy of the analysis of contralateral morphological MR images.

In summary, 2 years after surgery, knees with isolated MPFL reconstruction showed increased quantitative patellofemoral cartilage matrix degeneration and more severe knee joint degeneration as compared to the contralateral non-operated knee. Patients with patellar maltracking and increased patellar tilt seem to be at particular risk. MR imaging parameters indicating trochlear dysplasia and a postoperatively persistent high lateral patellar tilt were associated with worsening of knee joint degeneration after surgery. In conclusion, these findings indicate that isolated MPFL reconstruction successfully restores the deficient primary medial patellar soft tissue restraint, while a risk for progressive degenerative changes at the knee persists. Since patellar instability parameters influence the outcome after MPFL reconstruction, additional treatments in the case of borderline anatomic shapes may need to be discussed.

## Supplementary Information

Below is the link to the electronic supplementary material.

Supplementary file1 (PDF 4.78 MB)

## References

[1] Salonen EE, Magga T, Sillanpaa PJ, Kiekara T, Maenpaa H, Mattila VM (2017). Traumatic Patellar dislocation and cartilage injury: a follow-up study of long-term cartilage deterioration. Am J Sports Med.

[2] Snoeker B, Turkiewicz A, Magnusson K, Frobell R, Yu D, Peat G (2020). Risk of knee osteoarthritis after different types of knee injuries in young adults: a population-based cohort study. Br J Sports Med.

[3] Sanders TL, Pareek A, Johnson NR, Stuart MJ, Dahm DL, Krych AJ (2017). Patellofemoral arthritis after lateral patellar dislocation: a matched population-based analysis. Am J Sports Med.

[4] Baer MR, Macalena JA (2017). Medial patellofemoral ligament reconstruction: patient selection and perspectives. Orthop Res Rev.

[5] Previtali D, Milev SR, Pagliazzi G, Filardo G, Zaffagnini S, Candrian C (2020). Recurrent patellar dislocations without untreated predisposing factors: medial patellofemoral ligament reconstruction versus other medial soft-tissue surgical techniques-a meta-analysis. Arthroscopy.

[6] Erickson BJ, Nguyen J, Gasik K, Gruber S, Brady J, Shubin Stein BE (2019). Isolated medial patellofemoral ligament reconstruction for patellar instability regardless of tibial tubercle-trochlear groove distance and patellar height: outcomes at 1 and 2 years. Am J Sports Med.

[7] Erickson BJ, Mascarenhas R, Sayegh ET, Saltzman B, Verma NN, Bush-Joseph CA (2015). Does operative treatment of first-time patellar dislocations lead to increased patellofemoral stability? A systematic review of overlapping meta-analyses. Arthroscopy.

[8] Xing X, Shi H, Feng S (2020). Does surgical treatment produce better outcomes than conservative treatment for acute primary patellar dislocations? A meta-analysis of 10 randomized controlled trials. J Orthop Surg Res.

[9] Zhang K, Jiang H, Li J, Fu W (2020). Comparison between surgical and nonsurgical treatment for primary patellar dislocations in adolescents: a systematic review and meta-analysis of comparative studies. Orthop J Sports Med.

[10] Smith TO, Song F, Donell ST, Hing CB (2011). Operative versus non-operative management of patellar dislocation A meta-analysis. Knee Surg Sports Traumatol Arthrosc.

[11] Jungmann PM, Tham SC, Liebl H, Nevitt MC, McCulloch CE, Lynch J (2013). Association of trochlear dysplasia with degenerative abnormalities in the knee: data from the Osteoarthritis Initiative. Skeletal Radiol.

[12] Batailler C, Neyret P (2018). Trochlear dysplasia: imaging and treatment options. EFORT Open Rev.

[13] Wong TT, Denning J, Moy MP, Rasiej MJ, Redler LH, Ahmad CS, et al. MRI following medial patellofemoral ligament reconstruction: assessment of imaging features found with post-operative pain, arthritis, and graft failure. Skeletal Radiol. 2020. 10.1007/s00256-020-03655-x 33083857

[14] Kretzschmar M, Nevitt MC, Schwaiger BJ, Joseph GB, McCulloch CE, Link TM (2019). Spatial distribution and temporal progression of T2 relaxation time values in knee cartilage prior to the onset of cartilage lesions - data from the Osteoarthritis Initiative (OAI). Osteoarthritis Cartilage.

[15] Jungmann PM, Kraus MS, Nardo L, Liebl H, Alizai H, Joseph GB (2013). T(2) relaxation time measurements are limited in monitoring progression, once advanced cartilage defects at the knee occur: longitudinal data from the osteoarthritis initiative. J Magn Reson Imaging.

[16] Link TM, Li X (2018). Establishing compositional MRI of cartilage as a biomarker for clinical practice. Osteoarthritis Cartilage.

[17] Link TM, Neumann J, Li X (2017). Prestructural cartilage assessment using MRI. J Magn Reson Imaging.

[18] Baum T, Joseph GB, Karampinos DC, Jungmann PM, Link TM, Bauer JS (2013). Cartilage and meniscal T2 relaxation time as non-invasive biomarker for knee osteoarthritis and cartilage repair procedures. Osteoarthritis Cartilage.

[19] Banke IJ, Kohn LM, Meidinger G, Otto A, Hensler D, Beitzel K (2014). Combined trochleoplasty and MPFL reconstruction for treatment of chronic patellofemoral instability: a prospective minimum 2-year follow-up study. Knee Surg Sports Traumatol Arthrosc.

[20] Feucht MJ, Mehl J, Forkel P, Achtnich A, Schmitt A, Izadpanah K (2020). Failure analysis in patients with patellar redislocation after primary isolated medial patellofemoral ligament reconstruction. Orthop J Sports Med.

[21] Lutz PM, Winkler PW, Rupp MC, Geyer S, Imhoff AB, Feucht MJ. Complex patellofemoral reconstruction leads to improved physical and sexual activity in female patients suffering from chronic patellofemoral instability. Knee Surg Sports Traumatol Arthrosc. 2020. 10.1007/s00167-020-06340-7 PMC8384801 33119832

[22] Schottle PB, Hensler D, Imhoff AB (2010). Anatomical double-bundle MPFL reconstruction with an aperture fixation. Knee Surg Sports Traumatol Arthrosc.

[23] Kujala UM, Jaakkola LH, Koskinen SK, Taimela S, Hurme M, Nelimarkka O (1993). Scoring of patellofemoral disorders. Arthroscopy.

[24] Roos EM, Roos HP, Lohmander LS, Ekdahl C, Beynnon BD (1998). Knee Injury and Osteoarthritis Outcome Score (KOOS)–development of a self-administered outcome measure. J Orthop Sports Phys Ther.

[25] Dammerer D, Liebensteiner MC, Kujala UM, Emmanuel K, Kopf S, Dirisamer F (2018). Validation of the German version of the Kujala score in patients with patellofemoral instability: a prospective multi-centre study. Arch Orthop Trauma Surg.

[26] Gersing AS, Holwein C, Suchowierski J, Feuerriegel G, Gassert FT, Baum T (2020). Cartilage T2 relaxation times and subchondral trabecular bone parameters predict morphological outcome after matrix-associated autologous chondrocyte implantation with autologous bone grafting. Am J Sports Med.

[27] Allkemper T, Sagmeister F, Cicinnati V, Beckebaum S, Kooijman H, Kanthak C (2014). Evaluation of fibrotic liver disease with whole-liver T1rho MR imaging: a feasibility study at 1.5 T. Radiology.

[28] Peterfy CG, Schneider E, Nevitt M (2008). The osteoarthritis initiative: report on the design rationale for the magnetic resonance imaging protocol for the knee. Osteoarthritis Cartilage.

[29] Jungmann PM, Nevitt MC, Baum T, Liebl H, Nardo L, Liu F (2015). Relationship of unilateral total hip arthroplasty (THA) to contralateral and ipsilateral knee joint degeneration - a longitudinal 3T MRI study from the Osteoarthritis Initiative (OAI). Osteoarthritis Cartilage.

[30] Pfirrmann CW, Zanetti M, Romero J, Hodler J (2000). Femoral trochlear dysplasia: MR findings. Radiology.

[31] Dietrich TJ, Fucentese SF, Pfirrmann CW (2016). Imaging of individual anatomical risk factors for patellar instability. Semin Musculoskelet Radiol.

[32] Dejour DH (2013). The patellofemoral joint and its historical roots: the Lyon School of Knee Surgery. Knee Surg Sports Traumatol Arthrosc.

[33] Dejour H, Walch G, Nove-Josserand L, Guier C (1994). Factors of patellar instability: an anatomic radiographic study. Knee Surg Sports Traumatol Arthrosc.

[34] Lee PP, Chalian M, Carrino JA, Eng J, Chhabra A (2012). Multimodality correlations of patellar height measurement on X-ray, CT, and MRI. Skeletal Radiol.

[35] Dietrich TJ, Betz M, Pfirrmann CW, Koch PP, Fucentese SF (2014). End-stage extension of the knee and its influence on tibial tuberosity-trochlear groove distance (TTTG) in asymptomatic volunteers. Knee Surg Sports Traumatol Arthrosc.

[36] Macri EM, Culvenor AG, Morris HG, Whitehead TS, Russell TG, Khan KM (2018). Lateral displacement, sulcus angle and trochlear angle are associated with early patellofemoral osteoarthritis following anterior cruciate ligament reconstruction. Knee Surg Sports Traumatol Arthrosc.

[37] Macri EM, Felson DT, Zhang Y, Guermazi A, Roemer FW, Crossley KM (2017). Patellofemoral morphology and alignment: reference values and dose-response patterns for the relation to MRI features of patellofemoral osteoarthritis. Osteoarthritis Cartilage.

[38] Kumar D, Subburaj K, Lin W, Karampinos DC, McCulloch CE, Li X (2013). Quadriceps and hamstrings morphology is related to walking mechanics and knee cartilage MRI relaxation times in young adults. J Orthop Sports Phys Ther.

[39] Rauscher I, Stahl R, Cheng J, Li X, Huber MB, Luke A (2008). Meniscal measurements of T1rho and T2 at MR imaging in healthy subjects and patients with osteoarthritis. Radiology.

[40] Boelch SP, Gurok A, Gilbert F, Weissenberger M, Rudert M, Barthel T, et al. Why compromise the patella? Five-year follow-up results of medial patellofemoral ligament reconstruction with soft tissue patellar fixation. Int Orthop. 2021. 10.1007/s00264-020-04922-1 PMC8178154 33386924

[41] Fujii Y, Nakagawa S, Arai Y, Inoue H, Kan H, Hino M, et al. Clinical outcomes after medial patellofemoral ligament reconstruction: an analysis of changes in the patellofemoral joint alignment. Int Orthop. 2020. 10.1007/s00264-020-04765-w 32770307

[42] Tscholl PM, Wanivenhaus F, Centmaier-Molnar V, Camenzind RS, Fucentese SF (2020). Clinical and radiological results after one hundred fifteen MPFL reconstructions with or without tibial tubercle transfer in patients with recurrent patellar dislocation-a mean follow-up of 5.4 years. Int Orthop.

[43] Puzzitiello RN, Waterman B, Agarwalla A, Zuke W, Cole BJ, Verma NN (2019). Primary medial patellofemoral ligament repair versus reconstruction: rates and risk factors for instability recurrence in a young, active patient population. Arthroscopy.

[44] Matsushita T, Oka S, Araki D, Nishida K, Tanaka T, Kanzaki N (2017). Patient-based outcomes after medial patellofemoral ligament reconstruction. Int Orthop.

[45] Marcheggiani Muccioli GM, Lullini G, Grassi A, Macchiarola L, Cammisa E, Maccaferri B, et al. Good results are reported at 60-month follow-up after medial patello-femoral ligament reconstruction with fascia lata allograft for recurrent patellar dislocation. Knee Surg Sports Traumatol Arthrosc. 2020. 10.1007/s00167-020-06142-x 32651802

[46] Watkins LE, Rubin EB, Mazzoli V, Uhlrich SD, Desai AD, Black M (2020). Rapid volumetric gagCEST imaging of knee articular cartilage at 3 T: evaluation of improved dynamic range and an osteoarthritic population. NMR Biomed.

[47] Li AK, Pedoia V, Tanaka M, Souza RB, Ma CB, Li X (2020). Six-month post-surgical elevations in cartilage T1rho relaxation times are associated with functional performance 2 years after ACL reconstruction. J Orthop Res.

[48] Wang A, Pedoia V, Su F, Abramson E, Kretzschmar M, Nardo L (2016). MR T1rho and T2 of meniscus after acute anterior cruciate ligament injuries. Osteoarthritis Cartilage.

[49] Jibri Z, Jamieson P, Rakhra KS, Sampaio ML, Dervin G (2019). Patellar maltracking: an update on the diagnosis and treatment strategies. Insights Imaging.

[50] Weintraub S, Sebro R (2018). Superolateral Hoffa’s fat pad edema and trochlear sulcal angle are associated with isolated medial patellofemoral compartment osteoarthritis. Can Assoc Radiol J.

[51] van Middelkoop M, Macri EM, Eijkenboom JF, van der Heijden RA, Crossley KM, Bierma-Zeinstra SMA (2018). Are patellofemoral joint alignment and shape associated with structural magnetic resonance imaging abnormalities and symptoms among people with patellofemoral pain?. Am J Sports Med.

[52] Liao TC, Jergas H, Tibrewala R, Bahroos E, Link TM, Majumdar S, et al. Longitudinal analysis of the contribution of 3D patella and trochlear bone shape on patellofemoral joint osteoarthritic features. J Orthop Res. 2020. 10.1002/jor.24836 PMC8915432 32827327

[53] Haj-Mirzaian A, Guermazi A, Pishgar F, Roemer FW, Sereni C, Hakky M (2020). Patellofemoral morphology measurements and their associations with tibiofemoral osteoarthritis-related structural damage: exploratory analysis on the osteoarthritis initiative. Eur Radiol.

[54] Hefzy MS, Jackson WT, Saddemi SR, Hsieh YF (1992). Effects of tibial rotations on patellar tracking and patello-femoral contact areas. J Biomed Eng.

[55] Powers CM, Ward SR, Chan LD, Chen YJ, Terk MR (2004). The effect of bracing on patella alignment and patellofemoral joint contact area. Med Sci Sports Exerc.

[56] Hunter DJ, Zhang YQ, Niu JB, Felson DT, Kwoh K, Newman A (2007). Patella malalignment, pain and patellofemoral progression: the Health ABC Study. Osteoarthritis Cartilage.

[57] Ambra LF, Franciozi CE, Phan A, Faloppa F, Gomoll AH. Isolated MPTL reconstruction fails to restore lateral patellar stability when compared to MPFL reconstruction. Knee Surg Sports Traumatol Arthrosc. 2020. 10.1007/s00167-020-06015-3 32347346

[58] Sanchis-Alfonso V, Ginovart G, Alastruey-Lopez D, Montesinos-Berry E, Monllau JC, Alberich-Bayarri A, et al. Evaluation of patellar contact pressure changes after static versus dynamic medial patellofemoral ligament reconstructions using a finite element model. J Clin Med. 2019; 8(12). 10.3390/jcm8122093 PMC6947356 31805708

[59] Farr S, Huyer D, Sadoghi P, Kaipel M, Grill F, Ganger R (2014). Prevalence of osteoarthritis and clinical results after the Elmslie-Trillat procedure: a retrospective long-term follow-up. Int Orthop.

[60] Macri EM, Patterson BE, Crossley KM, Stefanik JJ, Guermazi A, Blomqwist E (2019). Does patellar alignment or trochlear morphology predict worsening of patellofemoral disease within the first 5 years after anterior cruciate ligament reconstruction?. Eur J Radiol.

[61] Zikria B, Rinaldi J, Guermazi A, Haj-Mirzaian A, Pishgar F, Roemer FW (2020). Lateral patellar tilt and its longitudinal association with patellofemoral osteoarthritis-related structural damage: analysis of the osteoarthritis initiative data. Knee.

[62] Eijkenboom JFA, van der Heijden RA, de Kanter JLM, Oei EH, Bierma-Zeinstra SMA, van Middelkoop M (2020). Patellofemoral alignment and geometry and early signs of osteoarthritis are associated in patellofemoral pain population. Scand J Med Sci Sports.

[63] Thuillier DU, Souza RB, Wu S, Luke A, Li X, Feeley BT (2013). T1rho imaging demonstrates early changes in the lateral patella in patients with patellofemoral pain and maltracking. Am J Sports Med.

[64] Liao TC, Martinez AGM, Pedoia V, Ma BC, Li X, Link TM (2020). Patellar malalignment is associated with patellofemoral lesions and cartilage relaxation times after hamstring autograft anterior cruciate ligament reconstruction. Am J Sports Med.

[65] Kim HK, Greenstein R, Plemmons A, Rajdev N, Parikh S, Kim DH. Patellofemoral instability in children: correlation between patellofemoral incongruence, mechanism of injury, and cartilage damage. AJR Am J Roentgenol. 2019:1–9. 10.2214/AJR.18.20778 30933650

[66] Maas KJ, Warncke M, Behzadi C, Welsch GH, Schoen G, Kaul MG (2020). Correlation of T2* relaxation times of the retropatellar cartilage with tibial tuberosity-trochlea groove distance in professional soccer players. Sci Rep.

[67] Nebelung S, Brill N, Tingart M, Pufe T, Kuhl C, Jahr H (2016). Quantitative OCT and MRI biomarkers for the differentiation of cartilage degeneration. Skeletal Radiol.

[68] Truhn D, Sondern B, Oehrl S, Tingart M, Knobe M, Merhof D (2019). Differentiation of human cartilage degeneration by functional MRI mapping-an ex vivo study. Eur Radiol.

[69] Balcarek P, Zimmermann F (2019). Deepening trochleoplasty and medial patellofemoral ligament reconstruction normalize patellotrochlear congruence in severe trochlear dysplasia. Bone Joint J.

[70] Nishioka H, Hirose J, Okamoto N, Okada T, Oka K, Taniwaki T (2015). Evaluation of the relationship between T1rho and T2 values and patella cartilage degeneration in patients of the same age group. Eur J Radiol.

[71] Jung M, Karampinos DC, Holwein C, Suchowierski J, Diallo TD, Gersing AS (2021). Quantitative 3-T magnetic resonance imaging after matrix-associated autologous chondrocyte implantation with autologous bone grafting of the knee: the importance of subchondral bone parameters. Am J Sports Med.

[72] Jungmann PM, Baum T, Bauer JS, Karampinos DC, Erdle B, Link TM (2014). Cartilage repair surgery: outcome evaluation by using noninvasive cartilage biomarkers based on quantitative MRI techniques?. Biomed Res Int.

[73] Jungmann PM, Gersing AS, Baumann F, Holwein C, Braun S, Neumann J (2019). Cartilage repair surgery prevents progression of knee degeneration. Knee Surg Sports Traumatol Arthrosc.

