# Supplementary material for: Patellar instability MRI measurements are associated with knee joint degeneration after reconstruction of the medial patellofemoral ligament
Source: Skeletal Radiol. 2021 Jul 4;51(3):535–47. doi: 10.1007/s00256-021-03832-6 (PMC8763754; doi:10.1007/s00256-021-03832-6)
Supplement: Supplementary file 1 — Supplementary file1 (PDF 4.78 MB) [file 256_2021_3832_MOESM1_ESM.pdf]

Supplementary material

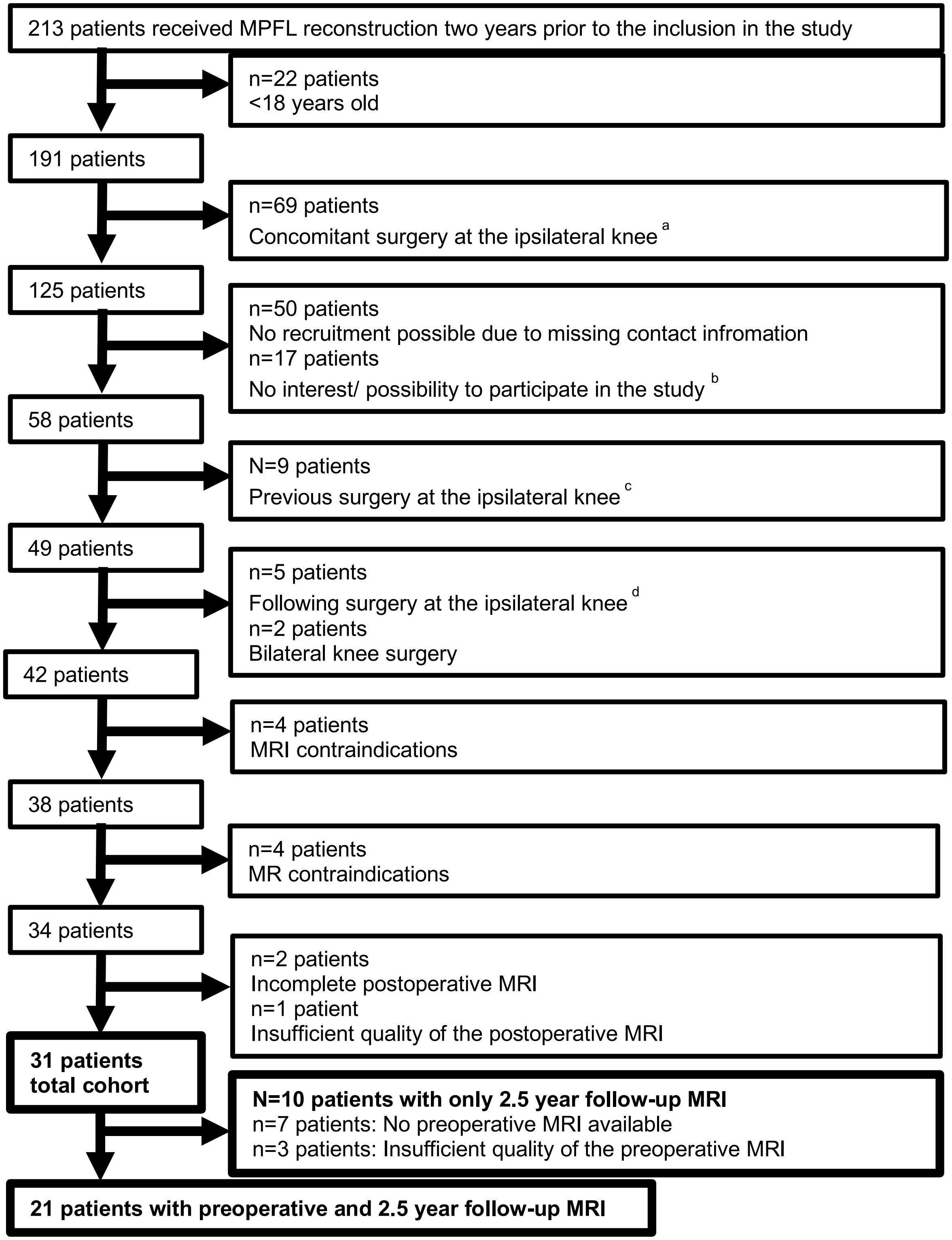

S1. Flowchart illustrating the patient selection.

WORMS Variable	Score	Classes
Menisci; Individual scoring for:		
Medial meniscus anterior horn	0	no lesion
Medial meniscus body	1	intrasubstance abnormalities
Medial meniscus posterior horn	2	non-displaced tear
Lateral meniscus anterior horn	3	displaced or complex tear without deformity
Lateral meniscus body	4	maceration
Lateral meniscus posterior horn		
Ligaments; Individual scoring for:		
Anterior cruciate ligament	0	none
Posterior cruciate ligament	1	signal abnormalities around Tendon/Ligament
Medial collateral ligament	2	signal abnormalities within the Tendon/Ligament
Lateral collateral lifament	3	partial tear
Patellar tendon	4	complete tear
Popliteal tendon		
Cartilage; Individual scoring for:	0	normal
Patella	1	signal abnormalities
Trochlea	2	partial thickness defect <1cm
Medial femoral condyle	2.5	sull thickness defect <1cm
Lateral femoral condyle	3	multiple partial <1cm, or partial defect ≥1cm (<75% of region)
Medial tibia	4	diffuse partial thickness loss > 75% of the region
Lateral tibia	5	multiple full thickness or full defect ≥1cm (<75% of region)
	6	diffuse full thickness loss > 75% of the regoin
Bone marrow edema; Individual scoring for:		
Patella	0	normal
Trochlea	1	minimal (minimal Ø <5 mm)
Medial femoral condyle	2	moderate (5≤ Ø <20 mm)
Lateral femoral condyle	3	severe (≥20 mm)
Medial tibia		
Lateral tibia		
Depression of surfaces; Individual scoring for:		
Patella	0	none
Trochlea	1	minimal
Medial femoral condyle	2	moderate
Lateral femoral condyle	3	severe
Medial tibia		
Lateral tibia		
Subarticular cysts; Individual scoring for:		
Patella	0	normal
Trochlea	1	minimal (minimal Ø <3 mm)
Medial femoral condyle	2	moderate (3≤ Ø <5 mm)
Lateral femoral condyle	3	severe (≥5 mm)
Medial tibia		
Lateral tibia		
Osteophytes; Individual scoring for:		
Patella	0	none
Trochlea	1	minimal
Medial femoral condyle	2	moderate
Lateral femoral condyle	3	severe
Medial tibia		
Lateral tibia		
Joint effusion	0	none
	1	mild (< 33% of maximum distention)
	2	moderate (33-66% of maximum distention)
	3	severe (> 66% of maximum distention)
Loose bodies	0	none
	1	1 loose body
	2	2 loose bodies
	3	>3 loose bodies
Popliteal cyst	0	normal
	1	mild
	2	moderate
	3	severe
**Maximum Total WORMS sum score**	**164**	

S2. WORMS scoring system.

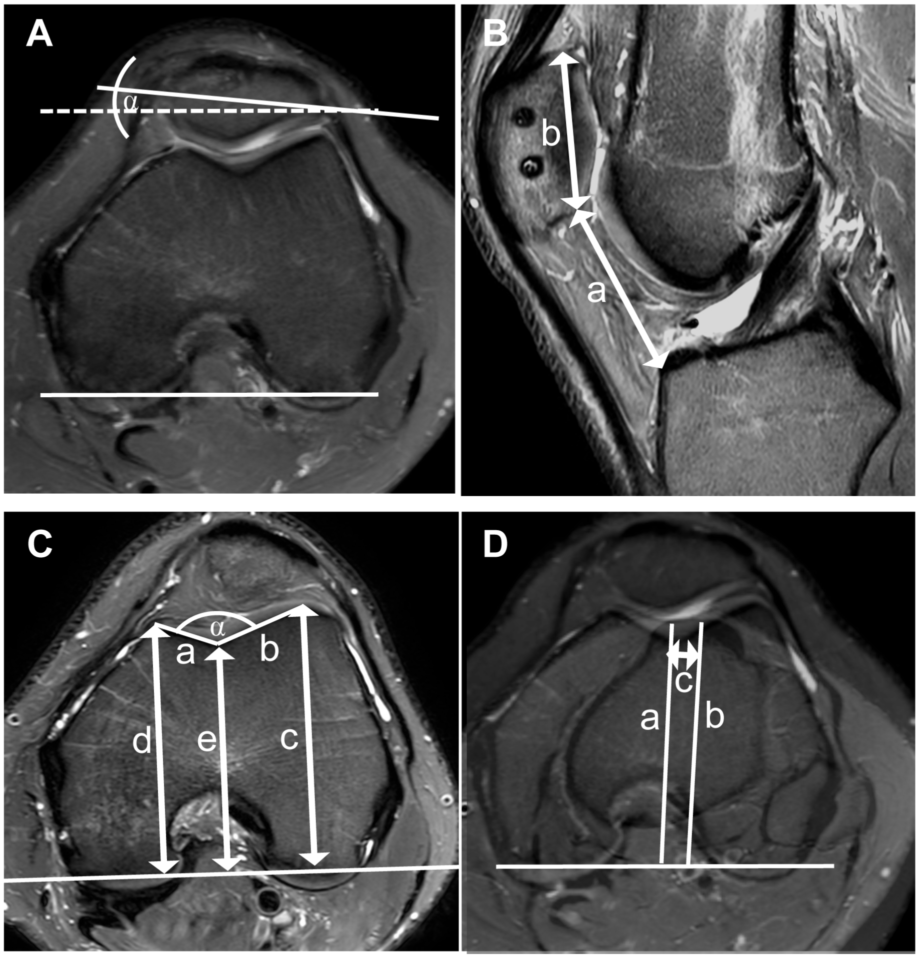

S3. Patellar instability measurements on MR imaging. Intermediate weighted MR images with fat saturation are shown. A: Lateral patellar tilt was determined by measuring the angle between a reference line through the patella and a line tangential along the femoral condyles posteriorly. B: Patellar height. The Caton-Dechamps index was calculated by dividing the distance of the lowest point of the patellar articular surface and the anterior point of the tibial plateau (a) by the length of the patellar articular length (b). C: Trochlear dysplasia measurements obtained at the transverse slice 30 mm proximal to the knee joint line. The angle between the medial and the lateral trochlear facet was measured to determine the sulcus angle (α). The facetal ratio was calculated as the ratio between the length of the medial trochlear facet (a) and the lateral trochlear facet (b). The trochlear depth was calculated as $[(c + d) / 2] - e$ while c represents the maximal distance from the lateral femoral condyle to the line tangential along the femoral condyles posteriorly, d represents the maximal distance from the medial femoral condyle to the line tangential along the femoral condyles posteriorly and e represents the minimal distance from the deepest point in the trochlear groove to the line tangential along the femoral condyles posteriorly. D: The tibial-tuberosity to trochlear groove (TTTG) distance was defined as the distance between the center of the tibial tuberosity and the deepest point of the trochlear groove on a line parallel to the line tangential along the femoral condyles posteriorly.

Parameter	Ipsilateral mean±SD (range)	Contralateral mean±SD (range)	P-value
Facetal ratio	0.49±0.12 (0.12 to 0.71)	0.54±0.14 (0.17 to 0.8)	0.051
Trochlear depth (mm)	4.7±1.4 (1.1 to 6.7)	5.3±1.7 (1.5 to 8.5)	**0.025***
Sulcus angle (°)	149±11° (127 to 177)	149±9° (129 to 172)	0.956
TTTG (mm)	13.3±4.0 (3.0 to 20.0)	10.0±4.2 (1.8 to 19)	**<0.001***
Patella tilt postOP (°)	13.16.2 (3 to 23.0)	14.7±7.7 (-6.5 to 27.3)	0.214
Caton Dechamps postOP	1.13±0.02 (0.89 to 1.42)	1.16±0.14 (0.96 to 1.45)	0.224

S4. Patellar instability measurements at the ipsilateral and at the contralateral knee. TTTG, tibial-tuberosity to trochlear groove distance; MR, magnetic resonance; postop, postoperatively; SD, standard deviation. *P<0.05
